# Tetramethylpyrazine (TMP), an Active Ingredient of Chinese Herb Medicine Chuanxiong, Attenuates the Degeneration of Trabecular Meshwork through SDF-1/CXCR4 Axis

**DOI:** 10.1371/journal.pone.0133055

**Published:** 2015-08-14

**Authors:** Na Yu, Zhang Zhang, Pei Chen, Yimin Zhong, Xiaoxiao Cai, Huan Hu, Ying Yang, Jing Zhang, Kaijing Li, Jian Ge, Keming Yu, Xing Liu, Jing Zhuang

**Affiliations:** State Key Laboratory of Ophthalmology, Zhongshan Ophthalmic Center, Sun Yat-sen University, Guangzhou, Guangdong, P. R. China; Duke University, UNITED STATES

## Abstract

**Background:**

A traditional Chinese medicine, Tetramethylpyrazine (TMP), has been prescribed as a complementary treatment for glaucoma to improve patient prognosis. However, the pharmacological mechanism of action of TMP is poorly understood. In previous studies, we demonstrated that TMP exerts potent inhibitory effects on neovascularization, suppresses the tumorigenic behavior of glioma cells, and protects neural cells by regulating CXCR4 expression. Here, we further investigated whether the SDF-1/CXCR4 pathway is also involved in the TMP-mediated activity in trabecular meshwork cells.

**Methodology/Principal Findings:**

CXCR4 expression was examined by quantitative real-time PCR in trabecular and iris specimens from 54 primary open-angle glaucoma (POAG) patients who required surgery and 19 non-glaucomatous donors. Our data revealed markedly elevated CXCR4 expression in the trabecular meshwork of POAG patients compared with that of controls. Consistently, CXCR4 expression was much higher in glaucomatous trabecular meshwork cells than in normal trabecular meshwork cells. Using RT-PCR and western blot assays, we determined that glaucoma-related cytokines and dexamethasone (DEX) also significantly up-regulated CXCR4 expression in primary human trabecular meshwork (PHTM) cells. Moreover, the TGF-β1-mediated induction of CXCR4 expression in PHTM cells was markedly down-regulated by TMP compared with control treatment (PBS) and the CXCR4 antagonist AMD3100. In addition, TMP could counteract the TGF-β1-induced effects on stress fiber accumulation and expansion of PHTM cells. TMP markedly suppressed the migration of PHTM cells stimulated by TGF-β1 in transwell and scratch wound assays. TMP also suppressed the extracellular matrix (ECM) accumulation induced by TGF-β2.

**Conclusions:**

Our findings demonstrate that CXCR4 might be involved in the pathogenetic changes in the trabecular meshwork of patients with POAG. Additionally, TMP might exert its beneficial effects in POAG patients by down-regulating CXCR4 expression.

## Introduction

Primary open-angle glaucoma (POAG), one of the leading causes of irreversible visual impairment worldwide [1], is characterized as a multi-factorial optic neuropathy [2–4]. Pathological ocular hypertension is the principle factor involved in the progression of optic neuropathy in POAG [5]. It is commonly initiated by impaired outflow of the aqueous humor and results from trabecular meshwork (TM) abnormalities, which include extracellular matrix (ECM) accumulation and structural changes in the actin cytoskeleton [6]. Currently, the molecular mechanisms underlying POAG are unclear, and the only well-established treatment involves lowering the intraocular pressure (IOP) [7]. However, symptomatic therapy alone is inefficient at controlling the progression of optic neuropathy in POAG. Chuanxiong was first described in the Chinese traditional medicine book Shennong Ben Cao Jing (a guide to Traditional Chinese Medicine), which was written in 200 BC, and the herb has been used to treat cardiovascular diseases for many years. 2,3,5,6-Tetramethylpyrazine (TMP), the bioactive component of Chuanxiong, has also been prescribed in the clinic as a complementary treatment for glaucoma to improve patient prognosis. Previous studies have reported that Chuanxiong therapy has a remarkable ability to control IOP in glaucoma patients, including via acupuncture injection, as a complementary treatment after surgery and in combination with other Chinese herbal medicines [8–10]. Moreover, Kitagawa K et al. reported that the IOP of eyes pretreated with TMP was significantly lower than that of controls in the acute high IOP pigmented rabbit model [11]. Although several scientific and clinical studies have provided solid evidence that TMP provides retinal neural protection and improves the prognosis of glaucoma patients [12–14], it remains unknown whether TMP can attenuate the pathological changes in the TM associated with POAG.

Chuanxiong, a multi-function traditional Chinese herb, has also been widely used to treat ischemia, cerebral infarction, degenerative diseases of the central nervous system, myocardial and pulmonary fibrosis and cancer with mild side effects [15–18]. Several studies have demonstrated that TMP effectively inhibits neovascularization, fibrosis and thrombosis under pathological conditions and suppresses C6 glioma growth [15, 19–21]. Moreover, our previous results indicated that TMP inhibits somatic Ca^2+^ increase in cerebral neurocytes and decreases the release of glutamate from glia cells through the SDF-1 (stromal cell-derived factor-1)/CXCR4 axis, thus providing neural protection [12]. Furthermore, TMP effectively inhibits the cell viability and migration of cultured C6 glioma cells by down-regulating CXCR4 expression. Importantly, the neurotoxicity caused by glutamate released from glioma cells is attenuated by TMP treatment, which reduces the damage to neural cells around the glioma cells [12]. These findings adequately account for previous discoveries [14, 22–23]. Prompted by these previous data, we demonstrated that TMP might inhibit corneal neovascularization, attenuate pulmonary fibrosis, and improve microcirculation by down-regulating CXCR4 expression both in vivo and in vitro [12, 15, 19]. However, little is known regarding the involvement of CXCR4 in the mechanisms of action of TMP related to glaucoma treatment.

CXCR4, a G protein-coupled receptor, is known to be the only receptor for SDF-1, which belongs to the CXC subfamily of chemokines. Unlike many chemokines whose expression is profoundly up-regulated during inflammatory responses, SDF-1 and its receptor CXCR4 are constitutively and widely expressed in various cell types throughout the human body [24]. Mounting evidence has indicated that the SDF-1/CXCR4 axis is involved in various fundamental processes, including neural system development during embryogenesis, ECM adhesion of hematopoietic cells, cell migration, and inflammatory and immune responses [25–27] Previous studies have demonstrated that the SDF-1/CXCR4 axis plays an important role in pathological angiogenesis and ocular inflammation [28]. In addition, De Groef et al. reported that matrix metalloproteinase (MMP) proteolysis, a key modulator of IOP homeostasis [29], is important in regulating chemokine activity [30]. Proteolytic processing of SDF-1 by MMP generates various amino-terminal truncated proteins that cannot interact with CXCR4 [30]. Moreover, several MMPs, including MMP-2, MMP-9, and MMP-13, directly or indirectly interact with CXCR4 and could thereby alter MMP-mediated pericellular proteolysis [31–32]. Therefore, we speculated that TMP not only protects optic nerves from apoptosis but also attenuates the degeneration of the TM in the treatment of POAG.

To evaluate this hypothesis, we examined CXCR4 expression levels in human TM specimens, TM cell lines and primary cultured TM cells. We observed abnormal up-regulation of CXCR4 in TM tissues and cells under pathological conditions. However, down-regulation of CXCR4 by TMP reduces the TGF-β-mediated degeneration of TM cells, with effects on cytoskeletal remodeling and ECM accumulation. In addition, TGF-β-stimulated migration was attenuated by TMP. Collectively, our study suggests that the pathological up-regulation of CXCR4 might be involved in the pathogenesis of TM degeneration in POAG. Furthermore, TMP might exert bioactivity in the clinical treatment of POAG by targeting the SDF-1/CXCR4 axis.

## Materials and Methods

### Human Trabecular Meshwork Specimens

Human TM and iris tissues were obtained from 54 POAG patients (15 to 60 years old) undergoing trabeculectomy with peripheral iridotomy for therapeutic purposes. The samples were processed less than 1 h after surgery. All the patients included in this study were diagnosed with POAG by Professor Liu Xing (Zhongshan Ophthalmic Center, Sun Yat-sen University) and presented with no other ocular pathologies or systemic diseases. Non-glaucomatous control TM and iris specimens were dissected from fresh human eye residuals (after corneal transplantation, within 24 h of enucleation) obtained from the eye banks of Zhongshan Ophthalmic Center (Guangzhou, China). This study was approved by the Ethical Review Committee of Zhongshan Ophthalmic Center at Sun Yat-sen University (Guangzhou, China), and this study adhered to the provisions of the Declaration of Helsinki for research involving human subjects. National ethics committee approval was obtained (approval #20131030), and all the patients signed the informed consent form before surgery.

### Patient Recruitment

Prior to surgery, clinical data for each patient were collected, including age, gender, use of prostaglandin analogs, number of argon laser trabeculoplasties and other ocular surgical interventions, type and duration of glaucoma, IOP, and visual acuity. Glaucoma diagnoses were based on careful clinical eye examinations, including slit lamp examinations, optical coherence tomography (OCT), gonioscopy, fundus photography and visual field examinations. All the patients included in this study required surgical treatment for medical reasons. The IOP exceeded 20 mmHg in all the patients in the glaucoma group at the time of surgery.

### Cell Culture and Treatment

Primary human trabecular meshwork (PHTM) cells (Cat. No. 6590, ScienCell, San Diego, CA, USA; see http://www.sciencellonline.com for details) were cultured in TM cell growth medium (TMCM, Cat. No. 6591, ScienCell), which contains basic medium (BM, ScienCell), 2% fetal bovine serum (FBS, Cat. No. 0010, ScienCell), 1% TM cell growth supplement (Cat. No. 6592, ScienCell) and 1% penicillin/streptomycin solution (P/S, Cat. No. 0503, ScienCell). The immortalized normal human trabecular cell line NTM was kindly provided by Professor Vincent Raymond (Laboratory of Ocular Genetics and Genomics, Quebec City, Canada), and the glaucomatous TM cell line GTM was a gift from Professor Yuhao Peng (Glaucoma Research; Alcon Laboratory, Fort Worth, TX) [33]. The cells were cultured in a humidified incubator with 95% air and 5% CO_2_ at 37°C. The cells were used from passages 4 to 12. All the experiments were performed at least three times.

The cells were treated with transforming growth factor (TGF)-β1 (5 ng/mL; Cat. No. 8915, CST, USA), TGF-β2 (5 ng/mL; Cat. No. T2815, Sigma, St. Louis, MO, USA), dexamethasone (DEX, 10^−7^ M; Cat. No. D4902, Sigma, St. Louis, MO, USA) or the appropriate vehicle control. AMD3100 (10 μg/mL) was purchased from Sigma (Cat. No. A5602, St. Louis, MO, USA). TMP hydrochloride (100 μM) was purchased from Harbin Medisan Pharmaceutical Co., China, and dissolved in normal saline to the appropriate concentrations.

### Real-time PCR

The mRNA expression of CXCR4 in human tissues and of ECM components in PHTM cells was assessed using a Roche 480 system and assays-on-demand primers for human CXCR4 and ECM components. Total RNA was isolated from TM cells using TRIzol Reagent (Invitrogen, USA). Reverse transcription-polymerase chain reaction (RT-PCR) assays were performed according to the manufacturer’s protocol for the SYBR Prime Script TM RT-PCR Kit (Takara, China). Real-time PCR was employed to measure the expression of CXCR4 and ECM components using the Roche 480 system (Roche, USA). Relative target gene expression was quantitated according to the comparative ΔCT method, i.e., normalized to an endogenous control gene, GAPDH, and relative to a calibrator after calculating the efficiency coefficient: relative expression = 2^-ΔCT^, where ΔCT = CT (target gene)-CT (GAPDH). The results are presented as the inverse of the normalized Ct value (InvCt) or as the relative fold change compared with an unstimulated control. The following primer pairs were used: CXCR4, 5’-TCTGGCAGCATGTTCTCTTC-3’ and 5’-CTCATTCCCACACTGGTGAC-3’; FN, 5’-AAGACCAGCAGAGGCATAAGG-3’ and 5’-TGTAGGGGTCAAAGCACGAG-3’; Col 1α, 5’-AGACAGTGATTGAATACAAAACCAC-3’ and 5’-AACGTCGAAGCCGAATTCC-3’; and GAPDH, 5’-GAGTCAACGGATTTGGTCGT-3’ and 5’-CATGGGTGGAATCATATTGGA-3’.

### Western Blotting

Cells were lysed with radio-immuno-precipitation assay buffer (50 mM Tris-HCl, pH 8.0, 150 mM sodium chloride, 1.0% IGEPAL CA-630 (NP-40), 0.5% sodium deoxycholate, and 0.1% sodium dodecyl sulfate) supplemented with a protease inhibitor cocktail. Total protein was extracted by centrifuging the tubes at 4°C for 15 min at maximum speed to remove debris. A total of 20 mg of protein was loaded into a sodium dodecyl sulfate/polyacrylamide electrophoresis gel for separation, and the proteins were then transferred for 1 h to a nitrocellulose (PVDF) membrane. CXCR4 was detected with a primary antibody against CXCR4 (1:100, Cat. No. ab2074, Abcam, USA) and a horseradish peroxidase-conjugated goat anti-rabbit secondary antibody (1:10000, Cat. No. 7074, CST, USA). GAPDH served as a loading control. Protein bands were detected using an enhanced chemiluminescence detection system (Millipore, USA).

### Immunofluorescence assay

Specimens and cells were fixed with 4% paraformaldehyde for 15 min. Slides were then immersed for 10 min in 0.1% Triton X-100 and blocked with 10% normal goat serum for 30 min. The cells were then incubated overnight at 4°C with primary antibodies against rabbit anti-CD45 (1:100, Cat. No. BA3371, Boster, China), rabbit anti-CXCR4 (1:100, Cat. No. ab2074, Abcam, USA), mouse anti- thrombospondin-1 (TSP-1) (1:100, Cat. No. ab1823, Abcam, Cambridge, MA) respectively. Secondary anti-mouse antibodies (1:500, Cat. No. 4408S, CST, USA) and anti-rabbit antibodies (1:500, Cat. No. 4413S, CST, USA) were added at room temperature, the nuclei were stained with DAPI. Images were captured by fluorescence microscopy.

### Immunohistochemical assay

Immunohistochemical assay was performed to identify PHTM cells according to the manufacturer’s protocols of SABC-AP kit (Cat. No. SA1052, Boster, China). Rabbit anti-fibronectin (FN) (1:100, Cat. No. BA1771, Boster, China), Rabbit anti-CD44 (1:100, Cat. No. BA0321, Boster, China), Rabbit anti-laminin (LN) (1:100, Cat. No. BA1762-1, Boster, China) were used as primary antibodies, and biotin-labeled goat anti-rabbit IgG antibodies as second antibody.

### Cell Viability Assay (MTT)

Human TM cells were incubated with MTT (5 mg/mL) for 4 h at 37°C, and then 150 μL of DMSO was added to each well in the 96-well plates. The absorbance was measured at 490 nm using a fluorescence plate reader (Power Wave XS; BIO-TEK). Cell viability was determined based on the optical density ratio of a treated culture relative to an untreated control.

### Actin polymerization assay

Phalloidin binds specifically to the F-actin polymer in mammalian cells, and this binding event was used to visualize the organization of F-actin in TM cells. PHTM cells were seeded on cover slips in 6-well plates. The cells were serum-starved overnight and then treated with 5 ng/mL TGF-β1 and phosphate-buffered saline (PBS), TMP (100 μM) or AMD3100 (10 μg/mL) for 24 h. To terminate the reaction, the cover slips were washed gently with PBS, fixed in 4% paraformaldehyde for 15 min and washed thrice with PBS. The cells on the cover slips were permeabilized for 10 min with 0.1% Triton X-100 and washed thrice with PBS. Subsequently, they were stained with FITC–phalloidin (Cat. No. P5282, Sigma, St Louis, MO, USA), a water-soluble compound that selectively binds to F-actin, for 2 h. Finally, the cover slips were washed thrice with PBS and mounted with an anti-fade reagent. Photomicrographs were captured with a ZEISS LSM 510 confocal microscope at 488 nm to obtain digital images. The staining fluorescence intensity of F-actin content as measured by LSM 510 examiner software was used to determine the stress-fiber formation.

### Cell migration assay

Cell migration was assessed using Transwell 24-well inserts (Corning Costar) with 8-μm pores. PHTM cells (1×10^5^) were suspended in 200 μL of serum-free medium and then added to the upper chambers of the transwells. The lower chambers contained 600 μL of medium containing 5% FBS. Each lower chamber contained TGF-β1, TGF-β1+AMD3100, TGF-β1+TMP or PBS. For the migration assays, the cells were incubated for 12 h. After 12 h, the non-migrated cells on the upper chambers were removed using cotton swabs; the cells on the lower chambers were fixed and stained for 15 min with 0.1% crystal violet (Beyotime, China), photographed (×40) and counted in 5 randomly selected fields for each membrane.

Wound healing assays were performed to quantify the rate of PHTM cell migration. A total of 1x10^6^ PHTM cells were seeded in a 60-mm dish and treated with TMP (100 μM), AMD3100 (10 μg/mL) or PBS with or without TGF-β1 (5 ng/mL) for 48 h. Then, a wound was created by manually scraping the cell monolayer with a P200 pipet tip. The initial wound was quantified using images collected at 0 h after wounding, when the wound size had stabilized. Additional images were collected at random wound areas at 12 and 24 h after wounding. Each sample was quantitatively analyzed using Image-Pro Plus software. The cell migration distance was determined by comparing the area of the wound under different experimental conditions to that under control conditions.

### Cell cycle assay

The PHTM cells were fixed with 75% ice-cold ethanol in PBS and stored at 4°C. Before analysis, the cells were washed twice with PBS and incubated for 30 min in a propidium iodide (PI) staining solution (0.05 mg/mL PI, 1 mM EDTA, 0.1% Triton-X-100 and 1 mg/mL ribonuclease A) (Sigma, St Louis, MO, USA). The fluorescence intensity was measured using a BD FACSort (BD Biosciences, USA) and was used to determine the G2/M ratio.

### Assessment of apoptosis

Apoptosis was quantified by flow cytometry using fluorescein isothiocyanate (FITC)-labeled Annexin V and PI (Annexin-V-PI Kit, Roche, Germany) according to the manufacturer’s protocols. The cell nuclei were stained with Hoechst dye. The cells were harvested with 0.02% trypsin after a 48-h incubation with TMP at different concentrations (0 μM, 200 μM or 400 μM). A minimum of 10,000 events were collected and analyzed using a FACS Calibur instrument and CellQuest Pro software (Becton Dickinson, USA).

### Statistical Analysis

All the in vitro experiments were performed at least in triplicate. The data are presented as the mean ± SD or median ± quartile. The differences between the means were evaluated using a two-tailed Student’s t-test (for two groups), analysis of variance (ANOVA, for more than two groups) or the Kruskal–Wallis test (for differences in CXCR4 levels). All the calculations and statistical tests were performed using SPSS (version 17.0; SPSS, Chicago, IL, USA). Differences with p<0.05 were considered significant in all the analyses.

## Results

### 1. CXCR4 is up-regulated in the trabecular meshwork in samples from POAG patients and in a glaucomatous cell line

**Fig 1:**
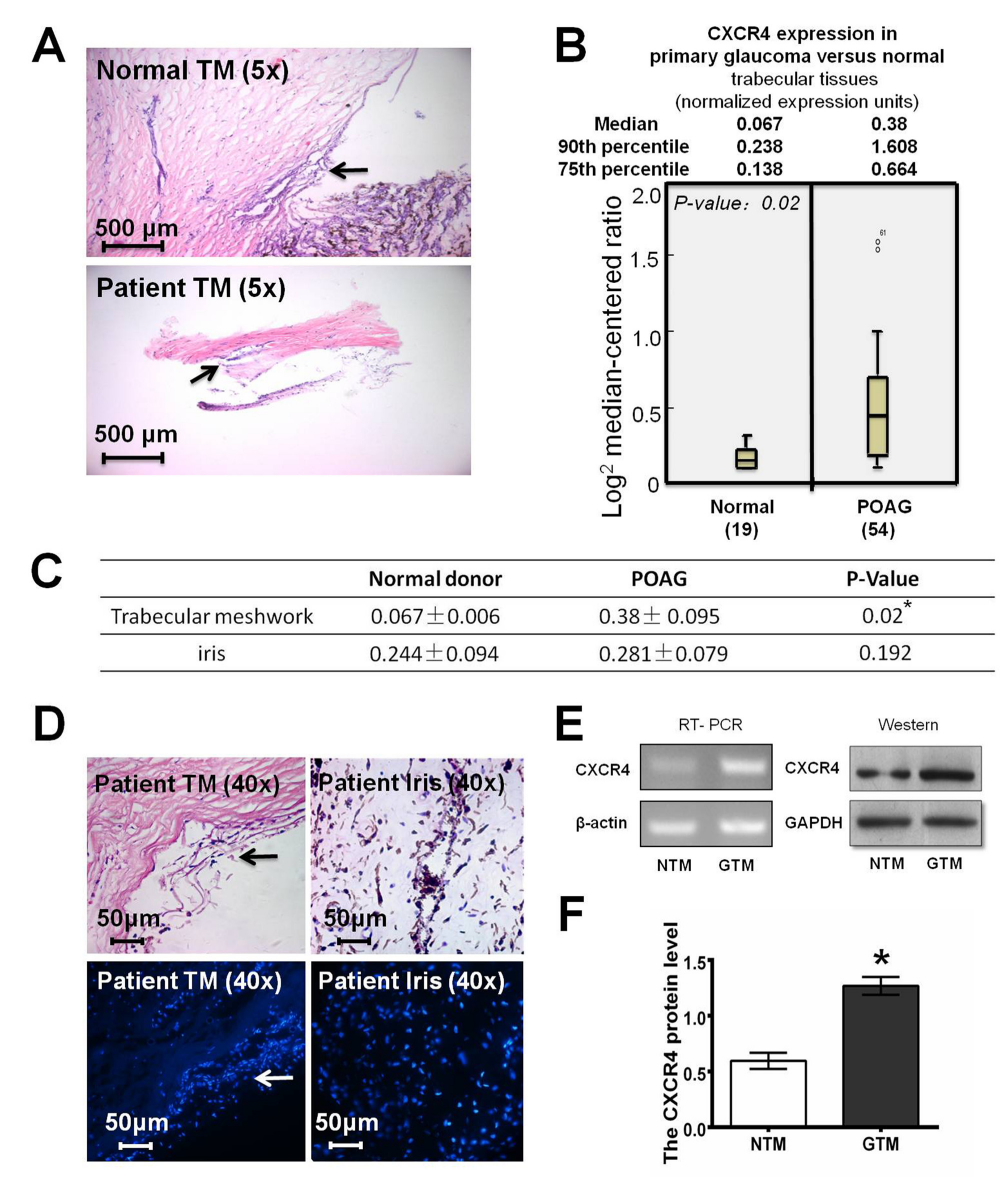
CXCR4 is up-regulated in the trabecular meshwork (TM) of primary glaucoma patients and of a glaucoma cell line. **A**, The histomorphology of human TM tissues from primary open-angle glaucoma (POAG) patients and non-glaucomatous donor controls was visualized by hematoxylin and eosin staining. **B,** CXCR4 expression levels in TM and iris specimens from 54 POAG patients and 19 non-glaucomatous controls were analyzed by real-time PCR. CXCR4 is weakly expressed in human TM tissues but is markedly up-regulated in TM tissues from POAG patients (0.067±0.006 versus 0.38±0.095, p = 0.02). **C,** CXCR4 expression in the TM and iris specimens from the two groups. CXCR4 expression in the iris was not significantly different between POAG patients and non-glaucomatous controls (0.244±0.094 versus 0.281±0.079, p = 0.192). The data are presented as the median ± quartile. **D,** Hematoxylin and eosin (H&E) staining and immunofluorescence for leukocyte common antigen (CD45) showed no inflammatory cell infiltrate in both TM and iris tissues of POAG patients. **E,** RT-PCR and Western blot analyses indicated that CXCR4 expression was higher in GTM cells than in NTM cells (CXCR4 mRNA expression: NTM, 0.672±0.109%; GTM, 1.121±0.188%; p<0.05. CXCR4 protein expression: NTM, 0.595±0.073%; GTM, 1.268±0.08%; p<0.05). β-actin or GAPDH was used as an internal loading control respectively. **F**, The relative quantification of CXCR4 expression in NTM and GTM cells was quantified by densitometry, and the data are presented as histograms. All the results were confirmed in three independent experiments. The error bars represent the standard deviation of the mean (n = 3). The asterisks indicate statistically significant differences between the control and experimental cells (*p<0.05).

To explore the bioactivity of CXCR4 in the pathogenesis of POAG, we first analyzed CXCR4 expression levels in trabecular and iris specimens from 54 POAG patients and 19 non-glaucomatous donors (Fig 1A). In the normal physiological state, CXCR4 is weakly expressed in the human TM tissues (Fig 1B). Additionally, CXCR4 mRNA expression is lower in the TM than in the iris (0.067±0.006 versus 0.244±0.094, Fig 1C). However, CXCR4 expression was markedly up-regulated in the TM of POAG patients compared with that of non-glaucomatous donors (0.067±0.006 versus 0.380±0.095, p = 0.027). Moreover, CXCR4 expression in the iris was not significantly different between POAG patients and non-glaucomatous controls (0.244±0.094 versus 0.281±0.079, p = 0.194). In addition, hematoxylin and eosin (H&E) staining and immunofluorescence for leukocyte common antigen (CD45) revealed no inflammatory cells in glaucomatous tissues (Fig 1D). These data suggest that the up-regulation of CXCR4 in the TM of POAG patients was not induced by inflammatory infiltration.

Additionally, we compared CXCR4 expression levels in a normal TM cell line (NTM) and a glaucomatous TM cell line (GTM). Consistent with our hypothesis, the relative quantification of CXCR4 expression (Fig 1E) in NTM cells (0.672±0.109%) was significantly lower than that in GTM cells (1.121±0.188%, p<0.05). Moreover, this result was confirmed by western blot analysis, which demonstrated that CXCR4 protein expression was higher in GTM cells (1.268±0.08%) compared with NTM cells (0.595±0.073%, p<0.05; Fig 1E and 1F). GAPDH was used as an internal loading control. Thus, we speculated that CXCR4 is involved in the pathogenesis of glaucoma rather than a result of inflammatory infiltration.

### 2. Glaucoma-related cytokines and dexamethasone induce the up-regulation of CXCR4 in primary trabecular meshwork cells

**Fig 2:**
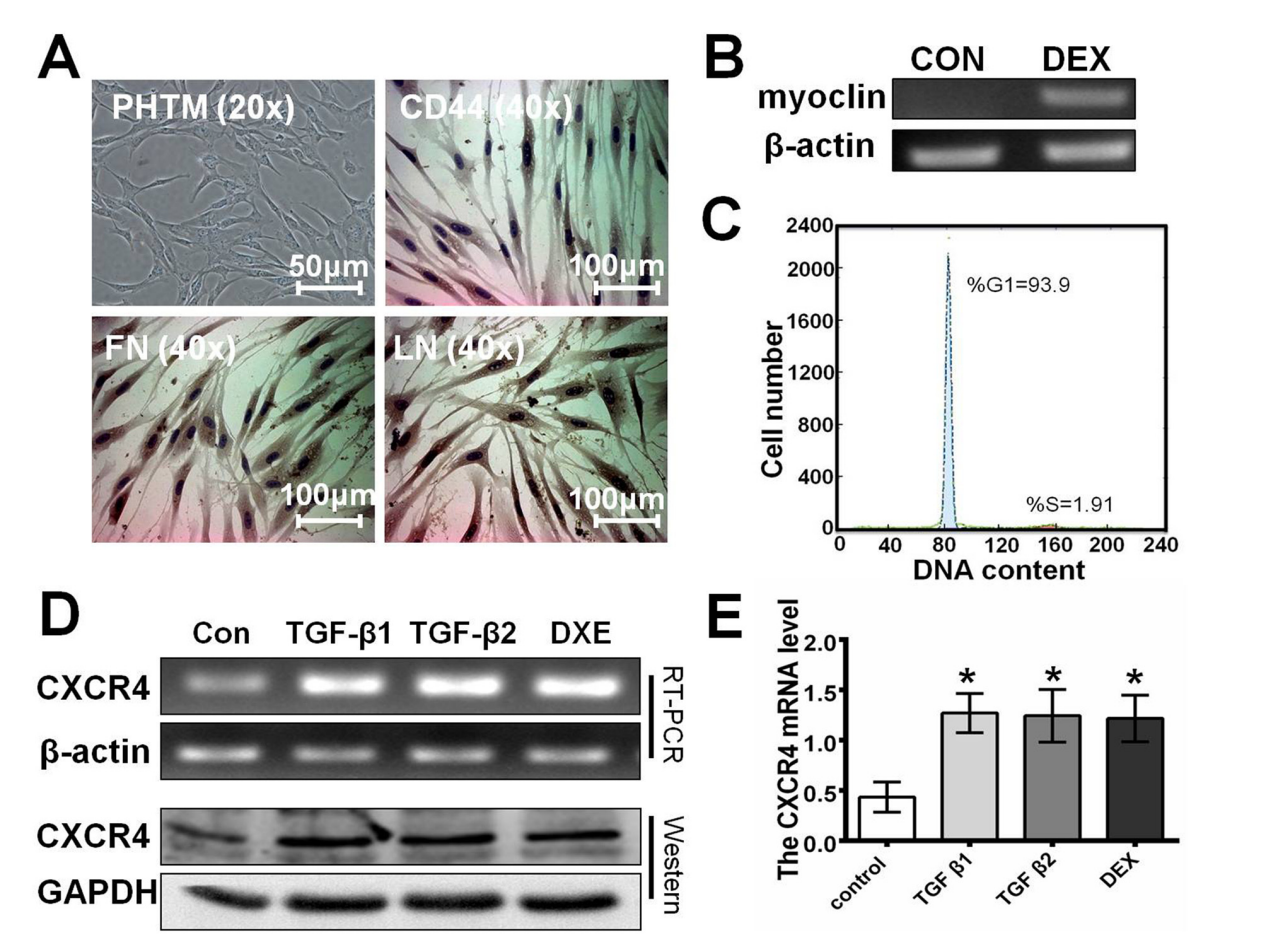
Glaucoma-related cytokines and DEX up-regulate CXCR4 in primary trabecular meshwork cells. **A**, Immunohistochemical staining analysis indicated that the primary cultured trabecular meshwork (PHTM) cells are positive for CD44, FN and LN. **B,** RT-PCR analysis indicated that myocilin expression in PHTM cells was strongly up-regulated after a 7-day incubation with dexamethasone (DEX; 0.392±0.134 vs. 1.461±0.307; p<0.01). **C,** The cell cycle analysis demonstrated that PHTM cells were dramatically arrested in G1 (G1: 94.37±3.45% and S: 3.43±0.06%), indicative of a non-dividing cell status. **D,** The RT-PCR and western blot analyses indicated that both the mRNA and protein levels of CXCR4 were markedly increased in PHTM cells after a 48-h incubation with TGF-β1, TGF-β2 or DEX (CXCR4 protein expression: Con, 0.076±0.014%; TGF-β1, 0.222±0.036%; TGF-β2, 0.164±0.031%; DEX, 0.155±0.025%; p<0.01. CXCR4 mRNA expression: Con, 0.435±0.151%; TGF-β1, 1.271±0.195%; TGF-β2, 1.244±0.261%; and DEX, 1.217±0.233%; p<0.01). β-actin or GAPDH was included as a loading control. **E,** The relative expression of CXCR4 in PHTM cells was quantified by densitometry, and the data are presented as histograms. All the results were confirmed in three independent experiments. The error bars represent the standard deviation of the mean (n = 3). The asterisks indicate statistically significant differences between the control and experimental cells (*p<0.01).

Immunohistochemical staining was performed to identify PHTM cells based on the expression of CD44, fibronectin (FN) and laminin (LN). As shown in Fig 2A, all the PHTM cells were strongly positive for FN, CD44 and LN. TGF-β1, TGF-β2 and DEX are principal pathological factors that are involved in the pathogenesis of glaucoma [34–37] The PHTM cells were characterized by increased myocilin expression after 7 days of DEX (10^−7^ M) treatment [37]. Therefore, PHTM cells were treated with DEX for 7 days. As shown in Fig 2B, the expression of myocilin in PHTM cells was low but was significantly up-regulated after 10 days of DEX treatment (0.392±0.134 and 1.461±0.307, respectively, p<0.01). In addition, the cell cycle analysis demonstrated that PHTM cells were dramatically arrested in G1 (G1: 94.37±3.45%; S: 3.43±0.06%), indicative of a non-dividing cell status (Fig 2C). Taken together, these results suggest the successful in vitro identification of PHTM cells.

Several active stressors, such as TGF-β, have been found in the aqueous humor of POAG patients and have been shown to induce morphologic and physiologic alterations in TM cells [34, 36]. To explore whether CXCR4 is involved in the pathogenesis of POAG, we performed RT-PCR and western blot assays to examine CXCR4 expression in PHTM cells after exposure to TGF-β1, TGF-β2 or DEX for 48 h. The RT-PCR and western blot analyses showed similar results (Fig 2D and 2E); CXCR4 mRNA and protein levels were markedly increased in PHTM cells after a 48-h incubation with TGF-β1, TGF-β2 or DEX (CXCR4 protein expression: Con, 0.076±0.014%; TGF-β1, 0.222±0.036%; TGF-β2, 0.164±0.031%; DEX, 0.155±0.025%; p<0.01. CXCR4 mRNA expression: Con, 0.435±0.151%; TGF-β1, 1.271±0.195%; TGF-β2, 1.244±0.261%; DEX, 1.217±0.233%; p<0.01). This evidence suggests that CXCR4 might be involved in the pathogenesis of POAG.

### 3. TMP inhibits CXCR4 expression in human primary trabecular meshwork cells after treatment with TGF-β

**Fig 3:**
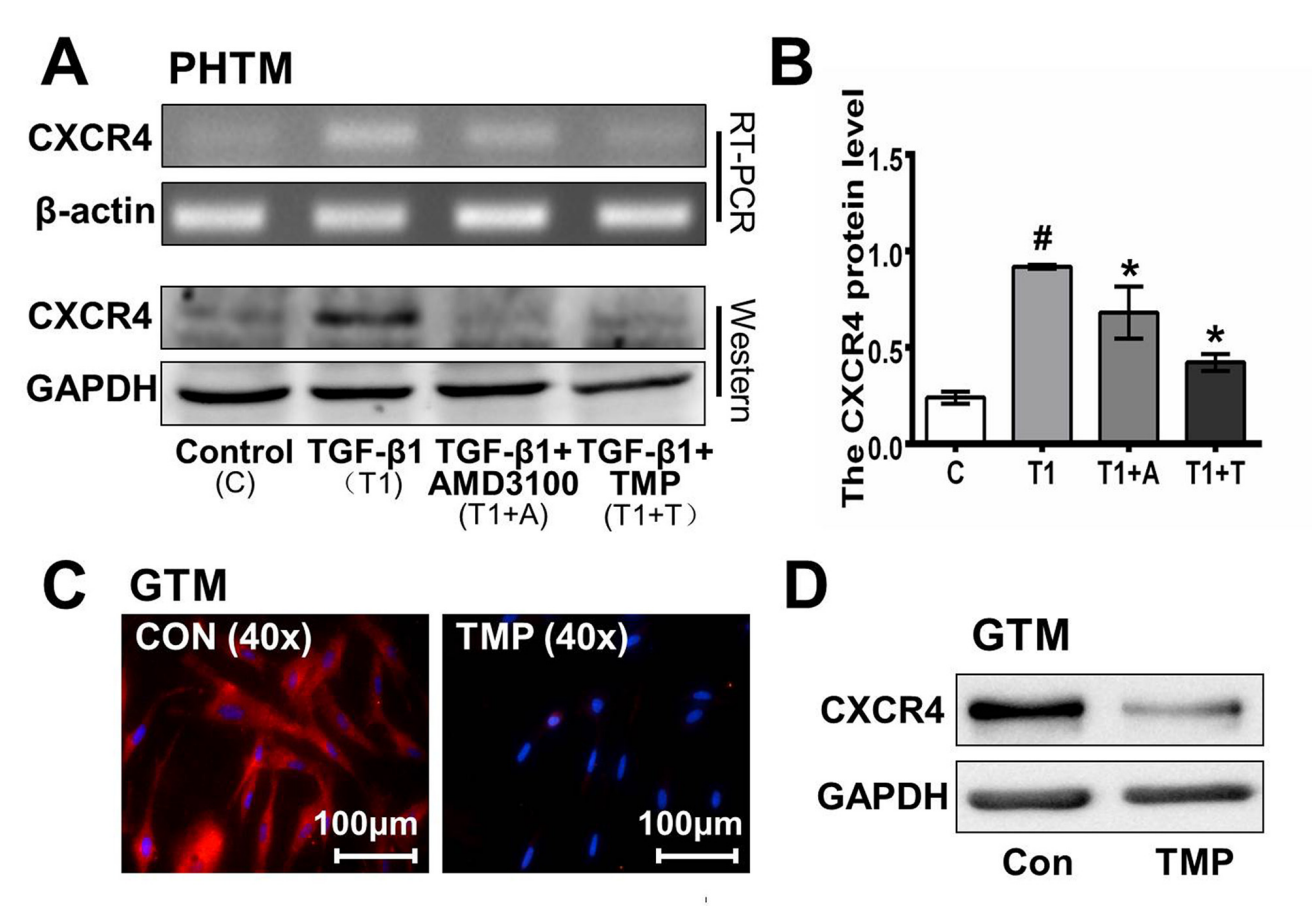
TMP inhibits CXCR4 expression in primary trabecular meshwork cells upon TGF-β1 treatment. **A,** Cells were treated with TGF-β1 (5 ng/mL) for 48 h in the presence of TMP (100 μM), AMD3100 (10 μg/mL) or PBS. RT-PCR and western blot analyses indicated that CXCR4 expression was up-regulated in PHTM cells by TGF-β1. However, this up-regulation was counteracted by TMP or AMD3100 (CXCR4 protein expression: Con, 0.24±0.031%; TGF-β1, 0.918±0.009%; TGF-β1+AMD3100, 0.681±0.135%; TGF-β1+TMP, 0.422±0.045%; p<0.01. CXCR4 mRNA expression: Con, 0.663±0.282%; TGF-β1, 1.192±0.195%; TGF-β1+AMD3100, 0.496±0.166%; TGF-β1+TMP, 0.434±0.037%; p<0.01). **B,** The relative expression of CXCR4 in PHTM cells was quantified by densitometry, and the data are presented graphically. **C,** Immunofluorescence staining revealed that CXCR4 expression in GTM cells was down-regulated by TMP. **D,** The western blot analysis indicated that CXCR4 protein expression in GTM cells was markedly down-regulated by TMP. All the results were confirmed in three independent experiments. The error bars represent the standard deviation of the mean (n = 3). #Statistically significant differences between the control and TGF-β1; *statistically significant differences between TGF-β1 and TGF-β1+TMP, TGF-β1+AMD3100 (#p<0.05; *p<0.05).

Our previous studies have demonstrated that TMP might inhibit cornea neovascularization, attenuate pulmonary fibrosis and suppress glioma cell behavior by regulating the SDF-1/CXCR4 axis [12, 15, 19]. Here, we observed that exogenous TGF-β1 (5 ng/mL) up-regulated CXCR4 expression in PHTM cells. To further investigate the bioactivity of TMP in POAG, RT-PCR and western blot assays were employed to examine CXCR4 expression in TGF-β1-treated PHTM cells in the presence of TMP (100 μM), AMD3100 (10 μg/mL, a specific inhibitor of CXCR4) or PBS. Treatment with TGF-β1 for 48 h significantly up-regulated CXCR4 expression in PHTM cells (Fig 3A and 3B). However, the up-regulation was significantly attenuated by TMP or AMD3100 (CXCR4 protein expression: Con, 0.240±0.031%; TGF-β1, 0.918±0.009%; TGF-β1+AMD3100, 0.681±0.135%; TGF-β1+TMP, 0.422±0.045%; p<0.01. CXCR4 mRNA expression: Con, 0.663±0.282%; TGF-β1, 1.192±0.195%; TGF-β1+AMD3100, 0.496±0.166%; TGF-β1+TMP, 0.434±0.037%; p<0.01). Moreover, we examined CXCR4 expression in GTM cells after TMP treatment by immunofluorescence and western blot. Consistent with our hypothesis, CXCR4 expression in GTM cells was significantly down-regulated by TMP (Fig 3C and 3D).

### 4. TMP attenuates TGF-β-mediated trabecular meshwork cell degeneration

**Fig 4:**
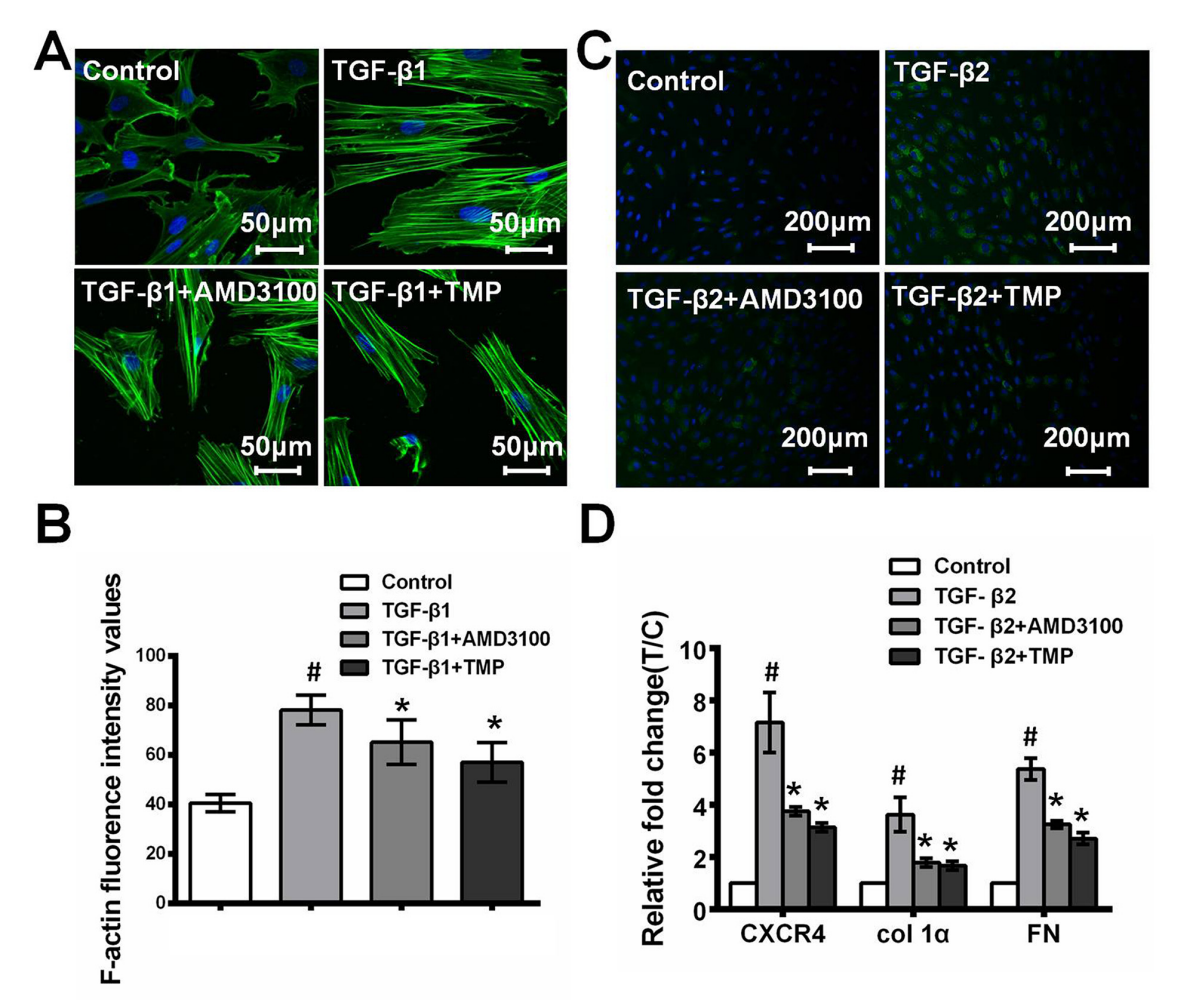
TMP attenuates TGF-β-mediate trabecular meshwork cells degeneration. **A,** TMP inhibits the TGF-β1-stimulated cytoskeleton arrangements in PHTM cells compared with AMD3100 treatment. The photomicrographs captured using a confocal microscope show that actin stress fibers and lamellipodial protrusions formed in PHTM cells upon TGF-β1 treatment, and this was inhibited by TMP and AMD3100. **B,** Images were captured by a confocal laser-scanning microscope (Zeiss LSM 510), and F-actin content was quantified using Zeiss LSM 510 examiner software to evaluate stress-fiber formation. F-actin content: Con: 40.50±3.45; TGF-β1: 78.17±6.08; TGF-β1+AMD3100: 65.17±9.06; TGF-β1+TMP, 57.00±8.05; #p<0.05, *p<0.05). **C,** Immunofluorescence for TSP-1 in cultured PHTM cells after treatment with TGF-β2 (5 ng/mL) for 48 h in the presence of TMP (100 μM) or AMD 3100 (10 μg/mL) or PBS. After treatment with TGF-β2, the intensity of staining for TSP-1 was considerably, an effect that was markedly reduced after treatment with TMP or AMD3100. **D,** Real-time PCR analyses indicated that the expression levels of CXCR4, FN and Col 1α were up-regulated in PHTM cells upon TGF-β2 treatment. However, this phenomenon was counteracted by TMP or AMD 3100 treatment (Relative fold change of CXCR4: TGF-β2, 7.16±1.16-fold; TGF-β2+AMD3100, 3.76±0.17-fold; TGF-β2+TMP, 3.14±0.17-fold; p<0.05. Relative fold change of Col 1α: TGF-β2, 3.63±0.67-fold; TGF-β2+AMD3100, 1.80±0.16-fold; TGF-β2+TMP, 1.68±0.16-fold; p<0.05. Relative fold change of FN: TGF-β2, 5.37±0.42-fold; TGF-β2+AMD3100, 3.25±0.15-fold; TGF-β2+TMP, 2.71±0.22-fold; p<0.05. All data are presented compared with the control). All results were confirmed in three independent experiments. The error bars represent standard deviation of the mean (n = 3). ^#^Statistically significant differences between the control and TGF-β; *statistically significant differences between TGF-β and TGF-β+TMP, TGF-β+AMD3100 (#p<0.05; *p<0.05).

The cytoskeleton of PHTM cells is known to be altered in glaucomatous eyes, and this has been hypothesized to reduce aqueous humor outflow [6]. Because TGF-β1 signaling has been considered as a critical determinant of actin cytoskeletal dynamics that contributes to the structural changes in TM cells in the pathology of primary glaucoma [38], cytoskeletal arrangements induced by TGF-β1 (5 ng/mL) were assessed using actin polymerization assays. Cells were serum-starved for 48 h in the presence of TMP (100 μM), AMD3100 (10 μg/mL) or PBS with or without TGF-β1. The accumulation of parallel actomyosin bundles (stress fibers) traversing the entire cell as well as cellular expansion were observed in PHTM cells treated with TGF-β1 compared with control cells (Fig 4A and 4B) (F-actin content: Con, 40.50±3.45; TGF-β1, 78.17±6.08; TGF-β1+AMD3100, 65.17±9.06; TGF-β1+TMP, 57.00±8.05; p<0.05). In addition, lamellipodial protrusions containing punctuate actin foci were observed. Interestingly, the expansion of PHTM cells induced by TGF-β1 was counteracted by TMP or AMD3100, as were the accumulation of actin stress fibers and the formation of lamellipodial protrusions. These observations suggest that TMP attenuates the TGF-β1-induced remodeling of the actin cytoskeleton in PHTM cells.

Previous studies have demonstrated the up-regulation of ECM components in the TM of POAG patients compared with normal TM. TGF-β2 has been associated with increased ECM deposition. To assess whether AMD3100 or TMP can reduce the TGF-β2-stimulated accumulation of ECM in PHTM cells, the cells were placed in culture plates and randomly divided into four groups: PBS, TGF-β2 (5 ng/mL), TGF-β2+AMD3100 (10 μg/mL), and TGF-β2+TMP (100 μM). The expression of ECM components was assessed by immunofluorescence staining assay and real-time PCR. As shown in Fig 4C, after treated with 5 ng/mL TGF-β2 for 48 h, a markedly increase TSP-1 was observed in PHTM cells, however, this phenomenon was attenuated by TMP or AMD3100. In addition, the up-regulation of CXCR4, FN and Col 1α in PHTM cells induced by TGF-β2 were decreased by TMP or AMD3100, as evidenced by real-time PCR, indicating that TMP suppresses the TGF-β2-mediated ECM accumulation (Relative fold change of CXCR4: TGF-β2, 7.16±1.16-fold; TGF-β2+AMD3100, 3.76±0.17-fold; TGF-β2+TMP, 3.14±0.17-fold; p<0.05. Relative fold change of Col 1α: TGF-β2, 3.63±0.67-fold; TGF-β2+AMD3100, 1.80±0.16-fold; TGF-β2+TMP, 1.68±0.16-fold; p<0.05. Relative fold change of FN: TGF-β2, 5.37±0.42-fold; TGF-β2+AMD3100, 3.25±0.15-fold; TGF-β2+TMP, 2.71±0.22-fold; p<0.05).

### 5. TMP inhibits the TGF-β-induced migration of human primary trabecular meshwork cells

**Fig 5:**
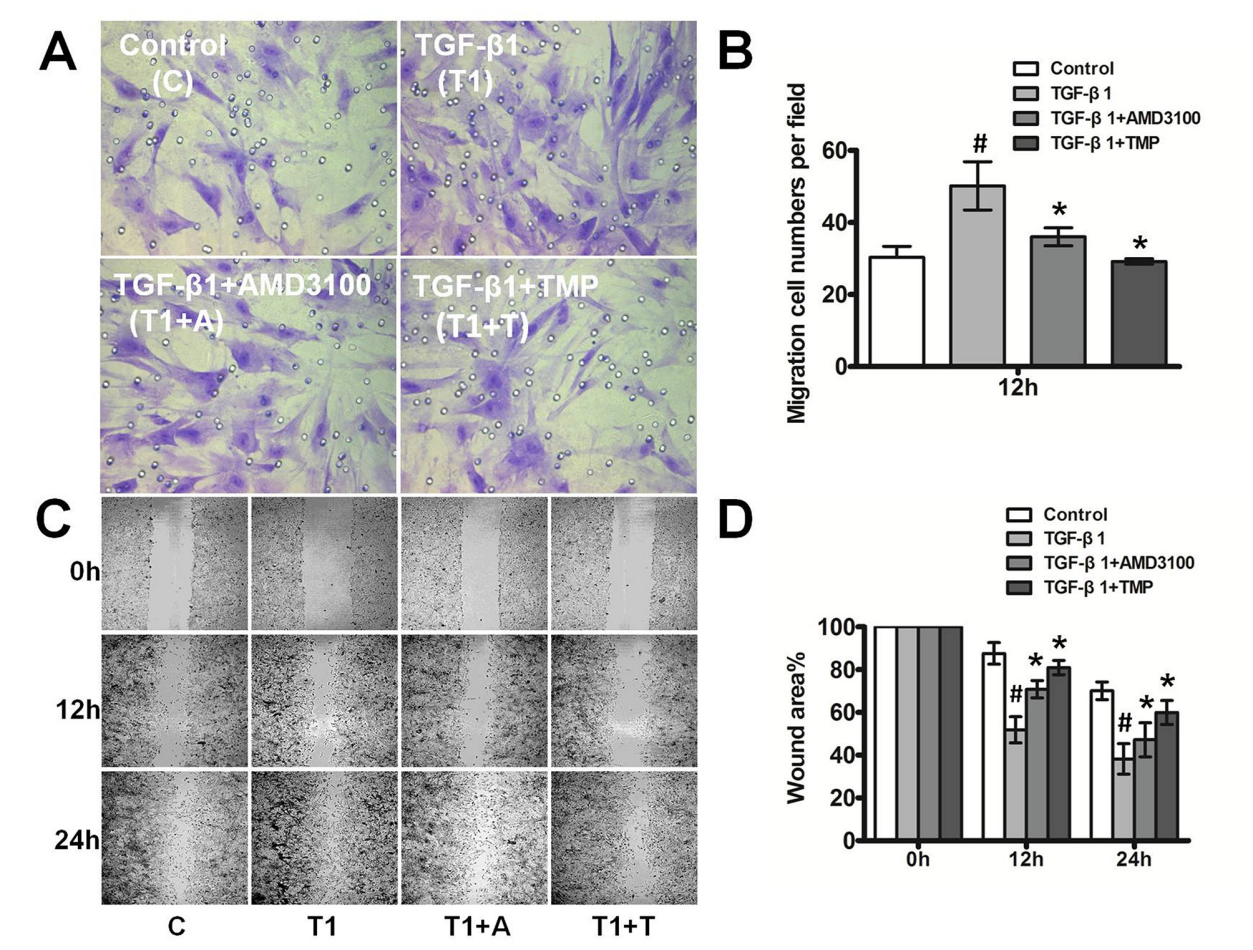
TMP inhibits migration in primary human trabecular meshwork cells after treatment with TGF-β1. A, The number of migrative cells was determined using transwell assay. The data showed that TGF-β1 significantly increased the cell migration speed and this effect was markedly reduced by TMP or AMD3100 treatment. B, Histogram representing the number of migrative cells per 40x field. Migration cell numbers per field: Con, 30.33±3.06; TGF-β1, 50.17±6.71; TGF-β1+AMD3100, 36.00±2.50; TGF-β1+TMP, 29.17±0.76; p<0.05. C, Representative phase contrast images demonstrating wound-induced PHTM cell migration at 12 h and 24 h. The scratch wound-healing assay indicated that TMP significantly decreased the migration of PHTM cells accelerated by TGF-β1, compared to the control. Further, the inhibition of cell migration by TMP was more effective than AMD3100. D, Histogram representing the relative wound area per 20x field. All results were confirmed in three independent experiments.

The effect of TMP on cell migration was evaluated using transwell assays, and the results were compared with those obtained from cells treated with AMD3100. Trypsinized cells were resuspended in serum-free medium and added to the upper chambers of transwell inserts; 600 μL of DMEM/F12 containing 5% FBS was added to the lower chambers. The cells were randomly divided into four groups: PBS, TGF-β1 (5 ng/mL), TGF-β1 (5 ng/mL)+AMD3100 (10 μg/mL), and TGF-β1 (5 ng/mL)+TMP (100 μM). After a 12- or 24-h incubation, the number of migrated cells was determined. PHTM cells treated with exogenous TGF-β1 migrated markedly faster than control cells, whereas inhibiting CXCR4 with AMD3100 or TMP decreased migration (Migration cell numbers: Con, 30.33±3.06; TGF-β1, 50.17±6.71; TGF-β1+AMD3100, 36.00±2.50; TGF-β1+TMP, 29.17±0.76; p<0.05) (Fig 5A and 5B).

The effect on PHTM cell migration was also assessed using the scratch wound healing assay. PHTM cells were plated in 6-well plates and divided into the same four groups as above. After a wound was created, the rapid proliferation of PHTM cells in the border zones led to rapid closure of the wound. As shown in Fig 5C and 5D, exogenous TGF-β1 markedly enhanced cell proliferation and migration, whereas this effect was significantly weakened by co-treatment with AMD3100 or TMP (12h, Con, 87.55±5.03%; TGF-β1, 51.80±6.13%; TGF-β1+AMD3100, 70.83±4.07%; TGF- β1+TMP, 80.95±3.33%; P <0.05. 24h, Con, 70.11±4.14%; TGF-β1, 38.22±7.12%; TGF-β1+AMD3100, 47.20±7.97%; TGF- β1+TMP, 59.96±5.57%; P <0.05). Moreover, inhibiting CXCR4 with TMP significantly decreased the migration of PHTM cells compared with AMD3100-mediated inhibition, as evidenced by the size of the remaining wound area.

### 6. TMP did not affect PHTM cell viability

**Fig 6:**
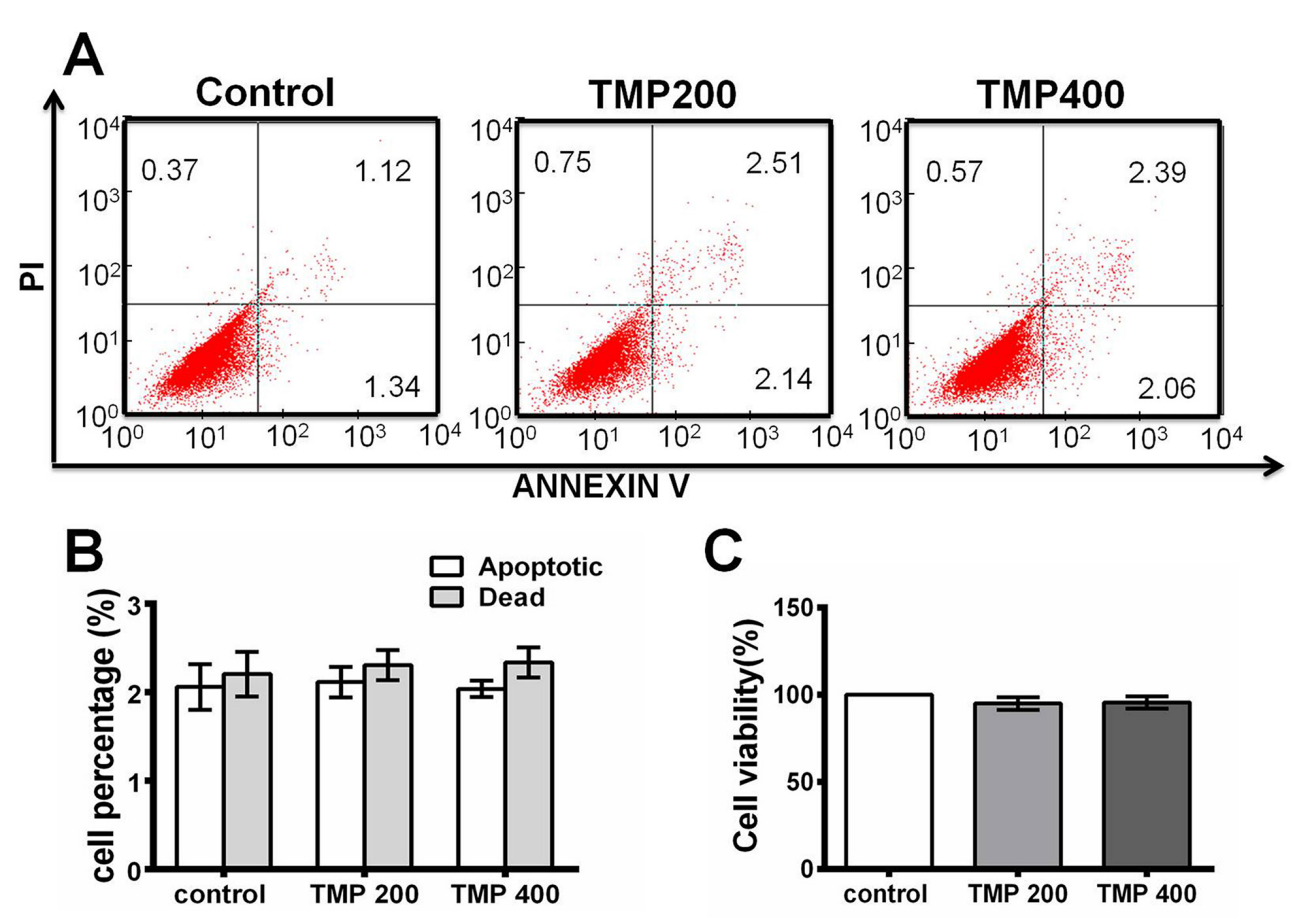
TMP did not affect the viability of PHTM cells. A, The early apoptosis of PHTM cells was quantified by flow cytometry after Annexin V and propidium iodide staining. The data showed that TMP had no effect on the incidence of early apoptosis in PHTM cells (Apoptosis: Control, 2.06±0.26%; TMP200, 2.11±0.172%; TMP400, 2.04±0.092%. Death: Control, 2.27±0.25%; TMP200, 2.31±0.171%; TMP400, 2.34±0.17%). B, The percentages of apoptotic and dead PHTM cells are presented in histograms. C, The viability of PHTM cells after treatment with TMP at different concentrations for 48 h was determined using MTT assays, and the data are presented as the percent survival compared with negative controls. TMP treatment did not affect the viability of PHTM cells. All the results were confirmed in three independent experiments. The error bars represent the standard deviation of the mean (n = 3). The asterisks indicate statistically significant differences between the control and experimental cells (*p<0.05).

To investigate the potential toxicity of TMP, the incidence of early apoptosis of PHTM cells was quantified by flow cytometry after Annexin V and PI staining. As shown in Fig 6A and 6B, TMP had no effect on the incidence of early apoptosis of PHTM cells (Apoptosis: Control, 2.06±0.26%; TMP200, 2.11±0.172%; TMP400, 2.04±0.092%. Death: Control, 2.27±0.25%; TMP200, 2.31±0.171%; TMP400, 2.34±0.17%; P>0.05). Additionally, cell viability after TMP treatment at different concentrations was assessed using MTT assays. Even at a high concentration (400 μM), TMP did not affect PHTM cell viability (95±3.68% and 95.73±3.49% compared with control after a 48-h incubation with TMP at 200 μM and 400 μM, respectively; P>0.05; Fig 6C), clearly indicating that TMP does not induce the apoptosis of PHTM cells.

## Discussion

TM degeneration has been generally considered as the principle factor involved in POAG. However, the underlying mechanism remains unknown. In the present study, we demonstrated that CXCR4, a G-protein-coupled receptor with 7 transmembrane-spanning domains, might be involved in the pathogenesis of POAG. We confirmed that TMP significantly down-regulates CXCR4 expression in GTM cells, suppresses TGF-β1-mediated cytoskeletal remodeling and migration and attenuated TGF-β2-mediated ECM accumulation in human TM cells. Thus, our present findings provide direct biochemical and functional evidence for the mechanism of action of a traditional Chinese herb in the clinical treatment of POAG.

The interaction between the chemokine receptor CXCR4 and its ligand SDF-1 performs multiple critical functions in normal and pathological physiology [39–40]. It has been reported that CXCR4 is widely expressed in different cell types in the hematopoietic, immune and nervous systems [41]. Here, our study revealed that CXCR4 expression is higher in TM specimens from glaucomatous patients than in those from normal human donors. Additionally, our data indicated that the over-expression of CXCR4 in TM tissues from POAG patients did not completely result from inflammatory infiltration. Moreover, the relative expression of CXCR4 was higher in the GTM cell line than in the NTM cell line. These results are consistent with those obtained in previous studies. Alvarado’s study indicated that cytokines play a key role in the homeostasis of aqueous humor outflow and potentially in the pathogenesis of glaucoma [42]. In vivo research has indicated that BAK enhances the induction of chemokines and elicits the degeneration of TM cells [43]. Alexandre reported that human glaucomatous TM tissues and human glaucomatous TM cell lines express chemokines and chemokine receptors, such as CXCR3 and CXCR4 [44]. Therefore, we hypothesized that the CXCR4 pathway might be involved in the pathogenesis of POAG.

The main glaucoma-related trabecular modification is characterized as age-related TM degeneration, which involves the accumulation of trabecular ECM and decreased cellularity and actin cytoskeletal dynamics in the TM [45]. Substantial evidence indicates that TGF-β signaling influences the structural changes in the actin cytoskeleton and the contractile properties of the TM in primary glaucoma [35–36]. Moreover, TGF-β is a well-known cytokine that has been implicated in the accumulation of ECM in TM tissues, thus resulting in reduced outflow facility in POAG patients [45]. However, the physiological mechanisms of TGF-β activation are not well understood. In this study, we observed a marked up-regulation of CXCR4 in PHTM cells after treatment with TGF-β1 or TGF-β2 for 48 h. Consistently, Bertran et al. reported that crosstalk exists between the TGF-β and CXCR4 pathways in pathological processes [46]. In particular, cytokines, chemokines and MMPs interact to coordinate ECM equilibrium [31–32]. Several MMPs, such as MMP-2 and MMP-9, directly or indirectly interact with CXCR4, which could alter the pericellular proteolysis of MMPs and result in the accumulation of ECM [31]. Taken together, our data suggest that CXCR4 might be involved in the pathogenesis of POAG.

The multiple pharmacological activities of TMP have been intensively researched, and TMP has shown promise in the complementary therapy of various ocular diseases, including corneal neovascularization and glaucoma. As described in previous studies, TMP might inhibit corneal angiogenesis and improve microcirculation by regulating the SDF-1/CXCR4 axis [15]. In the present study, we present evidence indicating that TMP down-regulates CXCR4 expression in the GTM cell line compared with controls. CXCR4 up-regulation in response to TGF-β1 in PHTM cells was notably suppressed by TMP. Moreover, our results demonstrated that TMP inhibits PHTM cell migration in the presence of TGF-β1 compared with the controls, indicating that TMP could alleviate the cellular burden induced by pathological factors.

More importantly, we found that TMP could counteract the cytoskeleton remodeling induced by TGF-β1, which corresponds with our previous study that demonstrated that TMP attenuates SDF-1-induced F-actin cytoskeleton remodeling in ECV304 cells [15]. The cytoskeleton of human TM cells is known to be altered in primary glaucomatous eyes, and it has been hypothesized to reduce aqueous humor through human TM tissue contraction [6]. When stimulated by stressor cytokines, such as TGF-β1, stress fibers and lamellipodial protrusions formed in PHTM cells, and the cells expanded. However, these phenomena were inhibited by TMP or AMD3100 (CXCR4 antagonist), suggesting that TMP might attenuate the pathological condition of TM through the SDF-1/CXCR4 axis. These results are consistent with those of previous studies. Liu et al. reported that CXCR4 significantly promotes angiogenesis in laser-induced choroidal neovascularization models and stimulates tube formation in and the migration of HRMECs through cytoskeleton remodeling [47–48]. Geraldine et al. also reported that CXCR4 inhibition results in a dramatic alteration in tip cell morphology and patterning [49]. In addition, ECM plays an important role in regulating IOP in both normal and glaucomatous eyes. Some previous studies have reported that TGF-β2 might be involved in the accumulation of ECM in the TM and cause resistance to TM outflow [45]. Our data revealed that in vitro stimulation with TGF-β2 promotes the expression of TSP-1, FN, Col 1α, CXCR4, but these effects were suppressed by TMP or AMD3100. In our previous studies, we demonstrated that TMP inhibits pathological angiogenesis in a rat model of corneal alkali burn injury, suppresses the migration of and tubule formation in ECV304 cells, improves microcirculation and suppresses tumors growth by regulating the SDF-1/CXCR4 axis [12, 15, 19]. Together with our previous results, our present study demonstrated that inhibiting CXCR4 expression with TMP alleviates the stimulatory effects of TGF-β and reduces the degeneration of TM cells, including cytoskeleton remodeling and ECM accumulation.

In addition, we performed MTT and cell apoptosis assays to evaluate the safety profile of TMP in PHTM cells. According to our data, TMP did not induce apoptosis in PHTM cells to any significant extent, which suggests its clinically acceptable safety profile. Moreover, several lines of evidence from in vitro and in vivo studies have suggested that TMP not only exerts a strong cytoprotective effect on photoreceptor cells and retina ganglion cells but also improves microcirculation and hemodynamic parameters, which could antagonize pathological progression in primary glaucoma patients [13, 15, 20–21]. Thus, our results suggest that TMP might partially improve the pathological condition of the TM by down-regulating CXCR4 expression.

In conclusion, our results identified CXCR4 over-expression in human glaucomatous tissues, a GTM cell line and TGF-β-treated PHTM cells, suggesting that CXCR4 might be involved in the pathogenesis of POAG. Additionally, TMP attenuates the TGF-β-induced pathological changes in the TM through the CXCR4 pathway, indicating that TMP is a multiple-functional medicine and a potential therapy for POAG. As a Chinese traditional herb, the clinical application of TMP is regionally limited because of the lack of clear recognition of its pharmacological mechanism of action. Therefore, the novel insights provided by the present study will extend the clinical therapeutic application of TMP in current medical practice.

## Supporting Information

S1 Fig The relative expression of CXCR4 in human tissues. The expression levels of CXCR4 of the trabecular and iris specimens in 54 primary open angle glaucoma patients and 19 non-glaucomatous controls were analyzed by real-time PCR (For trabecular meshwork: 0.067±0.006 versus 0.38±0.095, p = 0.02, For iris: 0.244±0.094 versus 0.281±0.079, p = 0.194).
(XLSX)

S2 Fig The scan of the purchase order of the primary human trabecular meshwork cell. (JPG)
