# Supplementary figures and images for: Tetramethylpyrazine (TMP), an Active Ingredient of Chinese Herb Medicine Chuanxiong, Attenuates the Degeneration of Trabecular Meshwork through SDF-1/CXCR4 Axis
Source: PLoS One. 2015 Aug 14;10(8):e0133055. doi: 10.1371/journal.pone.0133055 (PMC4537220; doi:10.1371/journal.pone.0133055)

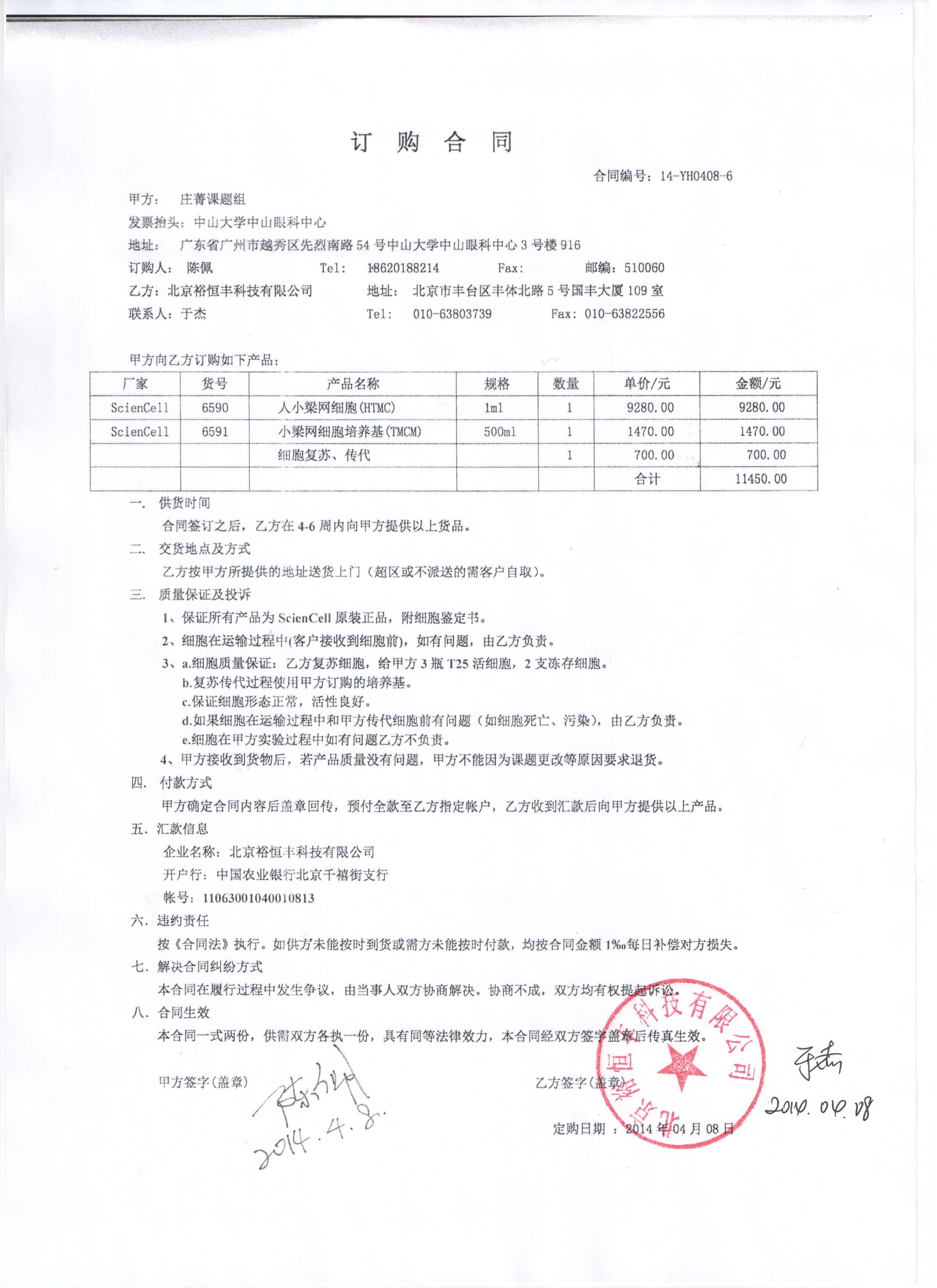

Supplement: S2 Fig — (JPG) [file pone.0133055.s002.jpg]
